# Marked reduction of alcohol dehydrogenase in keratoconus corneal fibroblasts

**Published:** 2009-04-10

**Authors:** V.V. Mootha, J.M. Kanoff, J. Shankardas, S. Dimitrijevich

**Affiliations:** 1 Department of Ophthalmology, University of Texas Southwestern Medical Center, Dallas, TX; 2 Integrative Physiology, University of North Texas Health Sciences Center, Fort Worth, TX

## Abstract

**Purpose:**

To identify differentially expressed genes in keratoconus (KC) corneal fibroblasts.

**Methods:**

Stromal keratocytes (having a fibroblast morphology) from KC keratoplasty specimens and eye bank donor corneas were isolated and expanded using a serum containing medium. RNA was isolated from three KC fibroblast cultures and five eye bank donor cornea fibroblast cultures. The targets from the cultured fibroblasts were hybridized to the Affymetrix U133 Plus 2.0 microarrays. Western blot analyses of cell lysates were performed to examine protein levels of interest in the two groups. Protein levels of select differentially expressed genes were further examined by immunohistochemistry. Keratocyte staining of archived KC keratoplasty specimens were graded using a 0 to 3+ scale and compared to five archived whole globes having normal corneas as well as to 10 Fuchs’ dystrophy keratoplasty specimens.

**Results:**

Microarray analysis revealed up to a 212 fold reduction in the mRNA levels of alcohol dehydrogenase (class 1) beta polypeptide (*ADH1B*) in KC fibroblasts (p=0.04). Decreased alcohol dehydrogenase in KC fibroblasts was confirmed by western blot analysis of early passage primary keratocyte cell lysates. Immunohistochemistry using a monoclonal mouse immunoglobulin G (IgG) against human liver alcohol dehydrogenase revealed a dramatic difference in protein staining in the keratocytes of the KC group compared to the normal cornea group. Immunohistochemistry also showed decreased immunostaining against alcohol dehydrogenase in the KC stromal sections compared to those obtained from Fuchs’ endothelial corneal dystrophy samples.

**Conclusions:**

Decreased alcohol dehydrogenase in KC corneal fibroblasts represents a strong marker and possible mediator of keratoconus.

## Introduction

Keratoconus (KC) is a non-inflammatory ectasia of the cornea that results in marked stromal thinning and irregular astigmatism. The incidence of the disorder is approximately 1 in 2,000 individuals in the general population. [1]. Patients may require penetrating or deep anterior lamellar keratoplasty procedures when rigid gas permeable contacts fail to provide functional vision. Collagen cross-linking with topical riboflavin and ultraviolet A irradiation is a promising treatment to stabilize the progressive stromal ectasia [2]. The histopathology of KC includes breaks in the epithelial basement membrane and Bowman’s membrane, marked central stromal thinning, anterior stromal scarring, iron in the basal epithelium (Fleischer ring), and breaks in Descemet’s membrane (hydrops). Keratocytes are responsible for the formation and maintenance of Bowman’s membrane and the stromal extracellular matrix.

The molecular pathogenesis of KC is poorly understood. Decreased corneal levels of protease inhibitors (such as alpha-1 proteinase inhibitor and alpha-2 macroglobulin) and increased levels of degradative enzymes may play a role in the stromal thinning [3-9]. Oxidative stress may also contribute to the pathogenesis of KC [10-19]. Keratoconus keratocytes (fibroblasts) have been shown to have increased production of reactive oxygen/nitrogen species, increased catalase activity, and higher levels of H_2_O_2_ compared to controls [16].

A genetic basis of the disease is supported by evidence of Mendelian inheritance in select families and by the association of KC with chromosomal abnormalities such as trisomy 21 [1]. Multiple gene loci probably contribute to the pathogenesis of KC based on preliminary genome-wide linkage analysis using sibling pair families [20]. Gene expression studies performed on KC keratoplasty corneal buttons provide a unique opportunity to delineate the molecular basis of the disorder. Lambiase et al. [21] reported the absence of nerve growth factor receptor (*TrkA*) mRNA in keratoconus and post-laser-assisted in situ keratomileusis (LASIK) ectasia buttons. Rabinowitz et al. [22] reported an absence of transcripts for aquaporin 5 using expressed sequence tag (EST) analysis of the cDNA library that was constructed from pooled KC keratoplasty buttons.

The recent advent of microarray based genomic analysis has facilitated a systematic discovery of molecular pathways impaired in human disease. Using microarrays to analyze the epithelium from KC patients, Nielsen et al. [23] found altered expressions of Charcot-Leyden crystal protein, desmoglein 3, epithelial membrane protein 3, S100 calcium binding protein A2, and secretory leukocyte peptidase inhibitor. Ha et al. [24] analyzed cultured KC corneal fibroblasts using a microarray containing 164 human apoptosis genes and found several that could play a role in the mechanism resulting in stromal thinning.

In our current study, we combined evidence from genome-wide expression profiling of KC corneal fibroblasts, western blot analysis of KC corneal fibroblast cell lysates, and immunohistochemistry of archived KC keratoplasty specimens to identify genes that may play a role in the pathogenesis of KC.

## Methods

### Corneal tissue

The study was approved by the Institutional Review Board at the University of Texas Southwestern Medical Center (Dallas, TX) and was performed to the tenets of the Declaration of Helsinki for research involving human subjects. In this study, 18 keratoconus corneas, 10 normal corneas, and 10 corneas with Fuchs’ endothelial dystrophy were used.

Five normal corneas and three KC corneas were used for microarray analysis. The normal corneas were donor corneas obtained from Florida Lions Eye Bank (Tampa, FL) transported in Optisol GS corneal storage medium (Bausch & Lomb, Rochester, NY) at 4 °C within 24 h post-mortem. The KC corneas were collected from cornea fellowship trained ophthalmologists within 24 h of the patient surgeries. The keratoplasty specimens were transported in Optisol GS (Bausch & Lomb) at 4 °C. The clinical diagnosis of KC was made by corneal specialists based on slit lamp findings (stromal thinning/ectasia, Fleischer ring, Vogt’s striae, and subepithelial/stromal scarring) and/or on Placido ring corneal topography signs.

Archived surgical keratoplasty and ocular pathology specimens (15 KC corneas, 5 normal corneas of whole globes enucleated for other pathology, and 10 Fuchs’ corneal endothelial dystrophy) were examined by immunohistochemistry. The histopathologic diagnosis of KC had been made by an ocular pathologist based on the presence of breaks in the epithelial basement membrane and Bowman’s membrane, central stromal thinning, anterior stromal scarring, iron in basal epithelium, and/or breaks in Descemet’s membrane. The histopathologic diagnosis of Fuchs’ dystrophy had been made by an ocular pathologist based on the presence of guttata and thickening of Descemet’s membrane with attenuation of the endothelium.

### Cell culture

Keratocytes (having a fibroblast morphology) from keratoconus keratoplasty specimens and eye bank donor corneas were isolated and expanded from stromal tissue using serum containing medium. Keratoplasty specimens were dissected in Hanks’ buffered saline solution (Life Technologies Inc., Grand Island, NY) to remove the epithelium and Descemet’s membrane with the endothelium. The stromal tissue was cut into 1 mm^3^ pieces and used as stromal explants for culture (at 37 °C in 5% CO_2_ humidified incubator) using Dulbecco’s modified Eagle’s medium containing 10% fetal bovine serum, 100 µg/ml penicillin, 100 µg/ml streptomycin, and 0.2 µg/ml amphotericin B (Life Technologies Inc.). Cells between passages 0 and 3 were used in this study.

### RNA isolation

Approximately 2×10^6^ cultured keratocytes were lysed for RNA isolation using Trizol^®^ reagent (Invitrogen, Carlsbad, CA) as per the manufacturer’s instructions. The cells were lysed in the tissue culture flask using 1 ml/cm^2^ Trizol^®^. The homogenate was then transferred to RNase free polypropylene tubes and incubated at room temperature for 5 min. Chloroform (0.5 ml to 1 ml of Trizol® used) was added to the homogenate, and the tubes were shaken vigorously and then incubated at room temperature for 5 min to allow complete mixing. The samples were then centrifuged at 12,000x g for 15 min at 4 °C. The aqueous phase (upper layer) containing the RNA was transferred to a fresh tube to which 0.5 ml of isopropanol/ml of Trizol^®^ was added. The samples were then centrifuged at 12,000x g for 10 min at 4 °C to pellet the RNA. The RNA pellet was then washed with 70% ethanol and dissolved in 50–100 μl of RNase free water and frozen at −80 °C for future use.

### Hybridization

Targets from cultured fibroblasts were hybridized to the Affymetrix U133 Plus 2.0 GeneChip microarray (Affymetrix Inc., Santa Clara, CA). Gene expression profiles were generated from three KC fibroblast cultures and five eye bank donor fibroblast cultures. The Affymetrix Microarray Suite Software (Affymetrix Inc.) was used to acquire and process the data including the evaluation of the abundance of each transcript. Gene expression levels were compared for fold change with the *t*-test used for statistical significance (Excel; Microsoft, Seattle, WA).

### Western blot analysis

Corneal fibroblast cells were treated with 300–600 μl lysis buffer (2.5 ml 1 M Tris buffer [pH=7.0], 1 g SDS, and 2.5 g sucrose in 50 ml distilled water) for 10 min. Genomic DNA was sheared by several passes (~10–20) through a 22 gauge needle, and samples were stored at –20 °C until needed. BCA protein assays (Pierce, Rockford, IL) of lysates were performed to ensure near equal loading of lanes. SDS-PAGE was performed at room temperature (RT) on 20 μg protein/lane using 4%–15% Tris-HCl Ready Gels (Bio-Rad, Hercules, CA) for 1 h and for 10 min at 95 V in Tris/glycine/SDS running buffer. Protein transfer onto nitrocellulose membranes was performed by electroblotting overnight (4 °C) at 25 mA in Tris/glycine buffer containing 20% methanol. Transfer was confirmed by Ponceau Red (Sigma-Aldrich Corp. St. Louis, MO) staining of the membranes. After de-staining in distilled water, membranes were blocked for 30 min (RT) in blocking buffer (5% powdered milk and 1% BSA in PBS) and blocking continued at 4 °C overnight followed by 30 min at room temperature the next morning. The solution was then discarded. The membranes were incubated with the primary antibody (ADH1B mouse polyclonal antibody raised against full length recombinant ADH1B [Abnova, Taipaei, Taiwan]) diluted in PBS overnight at 4 °C. Membranes were then rinsed three times, each time for 10 min in PBS and 0.1% Tween-20 and incubated with the secondary antibody for 1 h (RT). After rinsing three times, each time for 10 min in PBS and 0.1% Tween-20, membranes were developed **(**ECL chemiluminescence; Amersham Biosciences, Piscataway, NJ). Densitometric analysis of bands was performed using ImageJ image processing analysis in Java. Glyceraldehyde 3 phosphate dehydrogenase (GAPDH) was used as a loading control for normalization of experiments that were repeated three times.

### Immunohistochemistry

Immunohistochemistry was performed on five micron sections of archived surgical pathology cornea specimens that had been fixed in formalin and embedded in paraffin. A monoclonal mouse IgG against purified human liver alcohol dehydrogenase (Biogenesis/Morphosys Inc., Kingston, NH) was used. Sections were mounted on charged slides and deparaffinized and rehydrated through alcohol washes. Antigen retrieval was performed by microwaving slides for 7 min in citrate buffer. The sections were then incubated with the primary antibody at 4 °C overnight at a concentration of 12.6 µg/ml (1:30 dilution) in 2% BSA/PBS. Mouse IgG (BD PharMingen, San Jose, CA) at 12.6 µg/ml was used as a negative control. Slides were developed with 3,3′-diaminobenzidine (DAB; Vector Laboratories, Burlingame, CA) for 2 min and counterstained with 0.5% methyl green. The specimens were dehydrated and coverslipped with Permount (Thermo Fisher Scientific, Waltham, MA). Stromal keratocyte staining of five archived keratoconus keratoplasty buttons were graded in a masked fashion by two observers using a 0 to 3+ scale and were compared to five archived normal corneas of whole globes enucleated for posterior segment pathology. In a separate batch of immunohistochemistry experiments, the staining of stromal keratocytes in 10 archived keratoconus keratoplasty buttons were compared with the same in 10 archived Fuchs’ endothelial corneal dystrophy keratoplasty buttons.

## Results

### Microarray analysis

**Figure 1:**
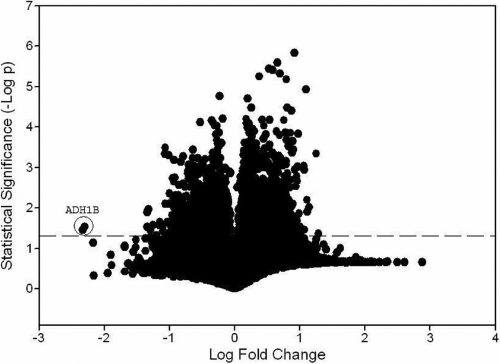
Fold change and p value data from microarray experiments. Encircled points represent transcripts for *ADH1B* (Probe sets 209612_s_at and 209613_s_at). Dashed line marks that p=0.05 and statistical significance.

Three different keratoconus keratoplasty specimens and five donor corneas from the eye bank, which acted as controls, were used for cell culture. The keratoconus specimens were from patients with a mean age of 25 years (age range: 18–29), and the mean age of the donor corneas from the eye bank was 24 years (age range: 17–30). Fold change and p value data from the microarray experiment are presented graphically as a volcano plot in Figure 1. Of over 54,000 probe sets analyzed with the Affymetrix platform, 3,900 were differentially expressed in the two groups using a p value of 0.05 for statistical significance. Marked decreases in alcohol dehydrogenase (class I) beta polypeptide (*ADH1B*), (class I) gamma polypeptide (*ADH1C*), and (class III) chi polypeptide (*ADH5*) were seen (Table 1). Microarray data analysis revealed up to a 212 fold reduction in the mRNA levels of alcohol dehydrogenase (class I) beta polypeptide (*ADH1B*) in the keratoconus fibroblasts (p=0.04). Raw expression levels of the *ADH1B* probe set are presented in Table 2. The *ADH1B* and *ADH5* raw probe set expression levels in the controls were robust enough to qualify for the “present” transcript abundance call by the Affymetrix Microarray Suite Software.

**Table 1:** Fold change of alcohol dehydrogenase gene family in keratoconus fibroblasts.

**Gene**	**Enzyme class**	**Probe Set**	**Fold change**	**p value**
*ADH1A*	I	207820_at	−1.8	0.23
*ADH1B*	I	209612_s_at	−212.5	0.04
		209613_s_at	−200.9	0.03
		209614_at	−148.2	0.07
*ADH1C*	I	206262_at	−20.8	0.01
*ADH4*	II	231678_s_at	−4.1	0.26
		231703_s_at	−3.5	0.46
		223781_x_at	1.1	0.75
		231675_s_at	1.2	0.66
*ADH5*	III	208848_at	−2.0	0.0006
		208847_s_at	−1.7	0.02
*ADH6*	V	214261_s_at	−1.10	0.88
		207544_s_at	1.7	0.39
*ADH7*	IV	210505_at	1.0	0.94

Statistically significant decreases in *ADH1B*, *ADH1C*, and *ADH5*  expression were found.

**Table 2:** Expression of *ADH1B* in keratoconus fibroblasts.

**Probe set ID**	**Keratoconus (n=3)**	**Eye Bank control (n=5)**	**Fold change**	**p value**
209612_s_at	114.67±180.04	24366.3±13426.45	−212.5	0.04
209613_s_at	73.37±65.06	14736.06±7719.59	−200.9	0.03
209614_at	18.1±7.82	2682.44±1834.46	−148.2	0.07

Raw expression values are mean±standard deviation. “n” indicates the number of different samples.

### Western blot analysis

**Figure 2:**
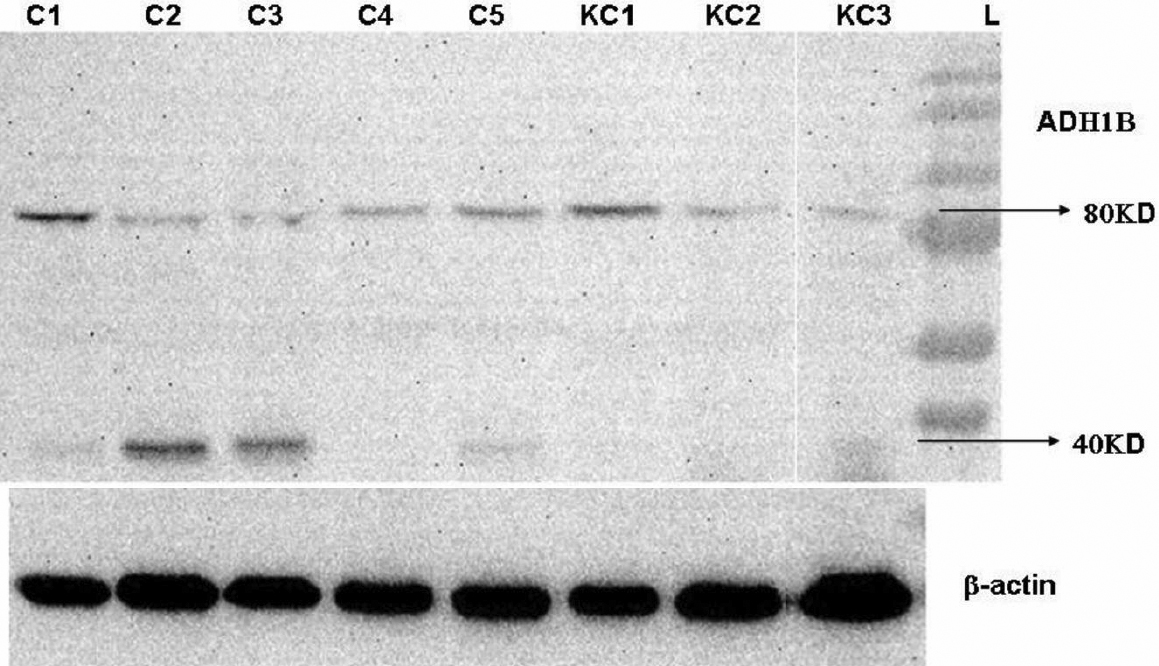
Protein expression of 80 kDa dimer and 40 kDa monomer subunits of alcohol dehydrogenase in cultured corneal fibroblasts. C1-C5 are normal corneal fibroblast cell lines, and K1–K3 are keratoconus corneal fibroblast cell lines.

Western blot analysis of the cell lysates of corneal fibroblasts using *ADH1B* mouse polyclonal antibody detected 40 kDa monomer subunits and 80 kDa dimer forms of the enzyme (Figure 2). Mean adjusted densitometry of the alcohol dehydrogenase (ADH) bands was 0.14 in the KC group versus 0.44 in the normal fibroblasts (p=0.03; Table 3).

**Table 3:** Normalized densitometry of alcohol dehydrogenase western blot bands.

**Specimen **	**Keratoconus fibroblasts**	**Normal fibroblasts**
Sample 1	0.22±0.03	0.6±0.03
Sample 2	0.09±0.01	0.59±0.03
Sample 3	0.11 ±0.2	0.49±0.01
Sample 4		0.22±0.03
Sample 5		0.32±0.01
Mean density*±SEM	0.14±0.04	0.44±0.07

The asterisk indicates that p=0.03. The western blots were repeated three times with each sample.

### Immunohistochemistry

**Figure 3:**
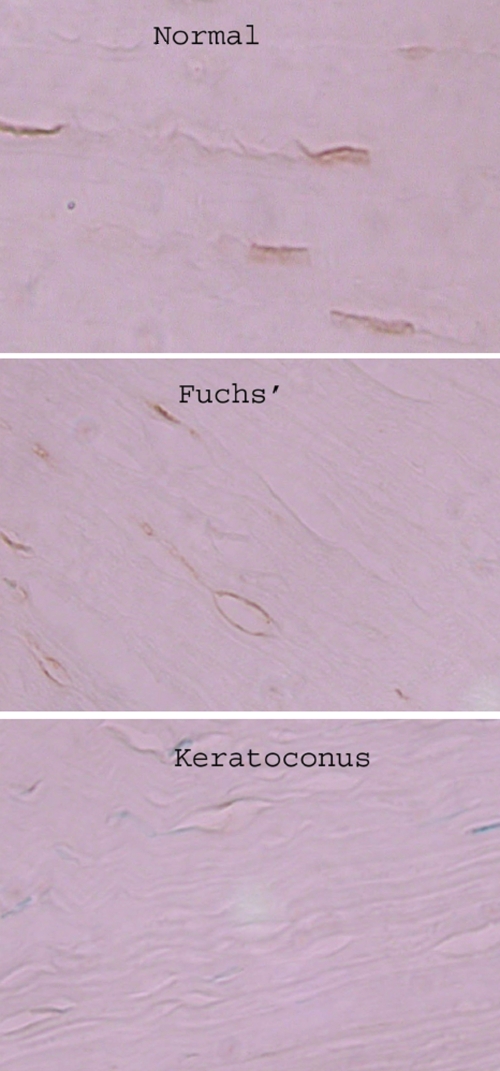
Alcohol dehydrogenase immunoreactivity in a normal cornea, Fuchs’ dystrophy cornea, and keratoconus cornea. Diaminobenzidine (DAB) brown staining of the keratocytes of normal corneas and Fuchs’ dystrophy corneas indicates presence of alcohol dehydrogenase in contrast to keratoconus keratocytes.

Immunohistochemistry experiments confirmed decreased protein levels of alcohol dehydrogenase in the stromal keratocytes of keratoconus corneas compared to normal corneas and Fuchs’ dystrophy corneas (Figure 3). Immunohistochemistry using a monoclonal mouse IgG against purified human liver alcohol dehydrogenase revealed a mean staining intensity of 0.1 in the stromal keratocytes in the keratoconus group compared to 1.4 in the normal cornea group (p=0.001; Table 4). In a separate series of immunohistochemistry experiments, there was a mean intensity of 0.6 in the stromal keratocytes of the keratoconus group compared to 1.7 in the Fuchs’ group (p=.005; Table 4).

**Table 4:** Mean intensity of alcohol dehydrogenase immunostaining of keratocytes in keratoconus keratoplasty specimens compared to normal corneas from enucleated whole globes and Fuchs’ keratoplasty specimens.

**Batch 1**	**Keratoconus (n=5)**	**Normal corneas (n=5)**	**p value**
	0.1±0.2	1.4±0.6	0.001
**Batch 2**	**Keratoconus (n=10)**	**Fuchs’ corneas (n=10)**	**p value**
	0.6±0.6	1.7±1.0	0.005

Immunostaining was graded using a 0 to 3+ scale. Values are mean±standard deviation.

## Discussion

**Figure 4:**
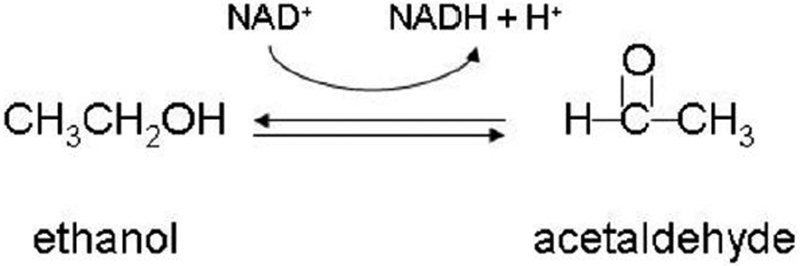
Alcohol dehydrogenase is a dimeric zinc metalloenzyme that catalyzes the reversible oxidation of alcohols to aldehydes.

*ADH1B* and *ADH1C* respectively code for the beta and gamma polypeptide of the class I human alcohol dehydrogenase enzyme. *ADH5* encodes for the chi polypeptide of the class III human alcohol dehydrogenase. Alcohol dehydrogenase (ADH) is a dimeric zinc metalloenzyme with 40 kDa subunits that catalyzes the reversible oxidation of alcohols to aldehydes (Figure 4) [25]. Enzymes within one class form homodimers and heterodimers with each other [25]. The *ADH* genes are expressed in a tissue specific pattern in the body and are important in detoxification pathways with the substrate ranging from methanol to long chain alcohols and sterols (retinol) [25].

The monoclonal mouse IgG against purified human liver alcohol dehydrogenase used in our immunohistochemistry experiments would be expected to stain multiple ADH isoenzymes encoded by *ADH1A*, *ADH1B*, *ADH1C*, *ADH4*, *ADH5*,**and *ADH6*. The ADH1B mouse polyclonal antibody used for the western blot experiments would be expected to additionally detect the gene products of *ADH1A *and* ADH1C* based on protein BLAST. The three class I ADHs are 94% identical in protein sequence, and there is a 60% sequence identity among different ADH classes [25]. The distribution, potential substrates, and functions of the various isoenzymes of alcohol dehydrogenase in the human cornea require further study.

Using a proteomics approach to identify the most abundant water soluble proteins in serum cultured human corneal fibroblasts, Karring et al. [26] reported the detection of alcohol dehydrogenase. This proteomics data taken together with our mRNA data, which show robust levels of *ADH1B* and *ADH5* transcripts in normal corneal fibroblasts, indicate that alcohol dehydrogenase is indeed important for normal corneal fibroblast physiology.

Julia et al. [27] reported significant presence of alcohol dehydrogenase (ADH) and aldehyde dehydrogenase (ALDH) in the rat cornea and suggested that these consecutive enzymes in the alcohol oxidation pathway may play a role in the detoxification of compounds produced by lipid peroxidation (high exposure to ultraviolet [UV] light and oxygen). Downes et al. [28] reported that treatment of adult male C57BL / 6J inbred mice with ultraviolet radiation (302 nm, 282 mW/cm^2^, for 1 h) resulted in a marked reduction of ADH and ALDH activity to 15%–16% of control animals with subsequent corneal clouding. Our finding of substantial reduction of alcohol dehydrogenase in keratoconus adds to the growing body of evidence that oxidative stress may contribute to the pathogenesis of KC [10-19].

Mouse knockout and other genetic studies have shown that ADH and ALDH play an important in vivo function in retinoid metabolism [29-31]. Retinoids are important for the regulation of normal growth, development, and cellular maintenance. Retinol (vitamin A) is a prerequisite for a normal conjunctival and corneal surface with deprivation resulting in xerophthalmia where there is a reduction of conjunctival goblet cells, in keratinization of the ocular surface, and in keratomalacia (stromal ulceration) [32]. In 1939, Mutch et al. [33] described keratoconus produced experimentally in the rat with vitamin A deprivation. Retinoic acid has been shown to inhibit expression of collagenase enzymes in cultured corneal fibroblasts from rabbits [34]. We can also speculate that a disturbance in ADH/ALDH homeostasis may disrupt the reaction of collagen aldehydes reacting with other collagen amino acids required in the intrinsic cross-linking process in the self-assembly of collagen fibrils [35].

Keratoconus research has been hampered by the difficulty of obtaining satisfactory corneal tissue for study from keratoconus patients and suitable control tissue. We cultured corneal fibroblasts from patients with advanced keratoconus requiring keratoplasty. We acknowledge that scarring and other effects of previous treatments such as rigid gas permeable contact lens use may alter gene and protein expression. Age-matched donor corneas from an eye bank were used to generate our control cell lines for the microarray and western blot experiments. To further validate our finding of decreased alcohol dehydrogenase expression in keratoconus, we performed immunohistochemistry experiments comparing archived keratoconus keratoplasty specimens to a small number of normal corneas from whole globes enucleated for posterior segment pathology. In a second batch of immunohistochemistry experiments, we were able to compare a larger number of archived keratoconus keratoplasty specimens to archived Fuchs’ dystrophy keratoplasty specimens that were readily available to us. The pathology of Fuchs’ dystrophy arises in the endothelial cell layer resulting in stromal edema with patients requiring keratoplasty much later in life than those patients with keratoconus. However, alcohol dehydrogenase levels were also markedly decreased in the stromal keratocytes in keratoconus compared to the unrelated corneal endothelial disorder of Fuchs’ dystrophy.

Our mRNA data suggest that the corneal fibroblast cell line is markedly altered in keratoconus. Although other proteomics studies have shown that alcohol dehydrogenase is highly expressed by normal corneal fibroblasts [26], this current study has shown that *ADH1B* is the most downregulated gene in keratoconus fibroblasts at the transcript level. Decreased levels of ADH protein in keratoconus fibroblasts are supported by our immunohistochemistry and western blot data. Given the importance of the enzyme in numerous metabolic pathways, we suggest that the absence of alcohol dehydrogenase is a robust marker and potentially a mediator of keratoconus. Further studies regarding the distribution and potential substrates of the alcohol dehydrogenase enzyme family in normal human cornea are required before we can understand the physiologic significance of reduced levels of ADH in keratoconus.

## References

[1] Rabinowitz YS (1998). Keratoconus.. Surv Ophthalmol.

[2] Wollensak G, Spoerl E, Seiler T (2003). Riboflavin/ultraviolet-A-induced collagen crosslinking for the treatment of keratoconus.. Am J Ophthalmol.

[3] Sawaguchi S, Yue BY, Sugar J, Gilboy JE (1989). Lysosomal enzyme abnormalities in keratoconus.. Arch Ophthalmol.

[4] Sawaguchi S, Twining SS, Yue BY, Wilson PM, Sugar J, Chan SK (1990). Alpha-1 proteinase inhibitor levels in keratoconus.. Exp Eye Res.

[5] Opbroek A, Kenney MC, Brown D (1993). Characterization of a human corneal metalloproteinase inhibitor (TIMP-1).. Curr Eye Res.

[6] Brown D, Chwa MM, Opbroek A, Kenney MC (1993). Keratoconus corneas: increased gelatinolytic activity appears after modification of inhibitors.. Curr Eye Res.

[7] Smith VA, Hoh HB, Littleton M, Easty DL (1995). Over-expression of a gelatinase A activity in keratoconus.. Eye.

[8] Zhou L, Sawaguchi S, Twining SS, Sugar J, Feder RS, Yue BY (1998). Expression of degradative enzymes and protease inhibitors in corneas with keratoconus.. Invest Ophthalmol Vis Sci.

[9] Smith VA, Easty DL (2000). Matrix metalloproteinase 2: involvement in keratoconus.. Eur J Ophthalmol.

[10] Gondhowiardjo TD, van Haeringen NJ, Volker-Dieben HJ, Beekhuis HW, Kok JH, van Rij G, Pels L, Kijlstra A (1993). Analysis of corneal aldehyde dehydrogenase patterns in pathologic corneas.. Cornea.

[11] Gondhowiardjo TD, van Haeringen NJ (1993). Corneal aldehyde dehydrogenase, glutathione reductase, and glutathione S-transferase in pathologic corneas.. Cornea.

[12] Buddi R, Lin B, Atilano SR, Zorapapel NC, Kenney MC, Brown DJ (2002). Evidence of oxidative stress in human corneal diseases.. J Histochem Cytochem.

[13] Behndig A, Karlsson K, Johansson BO, Brannstrom T, Marklund SL (2001). Superoxide dismutase isoenzymes in the normal and diseased human cornea.. Invest Ophthalmol Vis Sci.

[14] Kenney MC, Chwa M, Atilano SR, Tran A, Carballo M, Saghizadeh M, Vasiliou V, Adachi W, Brown DJ (2005). Increased levels of catalase and cathepsin V/L2 but decreased TIMP-1 in keratoconus corneas: evidence that oxidative stress plays a role in this disorder.. Invest Ophthalmol Vis Sci.

[15] Atilano SR, Coskun P, Chwa M, Jordan N, Reddy V, Le K, Wallace DC, Kenney MC (2005). Accumulation of mitochondrial DNA damage in keratoconus corneas.. Invest Ophthalmol Vis Sci.

[16] Chwa M, Atilano SR, Reddy V, Jordan N, Kim DW, Kenney MC (2006). Increased stress-induced generation of reactive oxygen species and apoptosis in human keratoconus fibroblasts.. Invest Ophthalmol Vis Sci.

[17] Udar N, Atilano SR, Brown DJ, Holguin B, Small K, Nesburn AB, Kenney MC (2006). SOD1: A candidate gene for keratoconus.. Invest Ophthalmol Vis Sci.

[18] Chwa M, Atilano SR, Hertzog D, Zheng H, Langberg J, Kim DW, Kenney MC (2008). Hypersensitive response to oxidative stress in keratoconus corneal fibroblasts.. Invest Ophthalmol Vis Sci.

[19] Olofsson EM, Marklund SL, Pedrosa-Domellof F, Behndig A (2007). Interleukin-1α downregulates extracellular-superoxide dismutase in human corneal keratoconus stromal cells.. Mol Vis.

[20] Li X, Rabinowitz YS, Tang YG, Picornell Y, Taylor KD, Hu M, Yang H (2006). Two-stage genome-wide linkage scan in keratoconus sib pair families.. Invest Ophthalmol Vis Sci.

[21] Lambiase A, Merlo D, Mollinari C, Bonini P, Rinaldi AM, D’Amato M, Micera A, Coassin M, Rama P, Bonini S, Garaci E (2005). Molecular basis for keratoconus: Lack of TrkA expression and its transcriptional repression by Sp3.. Proc Natl Acad Sci USA.

[22] Rabinowitz YS, Dong L, Wistow G (2005). Gene expression profile studies of human keratoconus cornea for NEIBank: a novel cornea-expressed gene and the absence of transcripts for aquaproin 5.. Invest Ophthalmol Vis Sci.

[23] Nielsen K, Heegard S, Vorum H, Birkenkamp-Demtroder K, Ehlers N, Orntoft TF (2005). Altered expression of CLC, DSG3, EMP3, S100A2, and SLPI in corneal epithelium from keratoconus patients.. Cornea.

[24] Ha NT, Nakayasu K, Murakami A, Ishidoh K, Kanai A (2004). Microarray analysis identified differentially expressed genes in keratocytes from keratoconus patients.. Curr Eye Res.

[25] Edenberg HJ (2000). Regulation of mammalian alcohol dehydrogenase genes.. Prog Nucleic Acid Res Mol Biol.

[26] Karring H, Thogersen IB, Klintworth GK, Enghild JJ, Moller-Pedersen T (2004). Proteomic analysis of the soluble fraction from human corneal fibroblasts with reference to ocular transparency.. Mol Cell Proteomics.

[27] Julia P, Farres J, Pares X (1986). Ocular alcohol dehydrogenase in the rat: regional distribution and kinetics of the ADH1-isoenzyme with retinol and retinal.. Exp Eye Res.

[28] Downes JE, Swann PG, Holmes RS (1993). Ultraviolet light induced pathology in the eye: associated changes in ocular aldehyde dehydrogenase and alcohol dehydrogenase activities.. Cornea.

[29] Duester G (2001). Genetic dissection of retinoid dehydrogenases.. Chem Biol Interact.

[30] Molotkov A, Duester G (2003). Genetic evidence that retinaldehyde dehydrogenase Raldh1 (Aldh1a1) functions downstream of alcohol dehydrogenase Adh1 in metabolism of retinol to retinoic acid.. J Biol Chem.

[31] Pares X, Farres J, Kedishvili N, Duester G (2008). Medium- and short-chain dehydrogenase/reductase gene and protein families: Medium-chain and short-chain dehydrogenases/reductases in retinoid metabolism.. Cell Mol Life Sci.

[32] Hatchell DL, Sommer A (1984). Detection of ocular surface abnormalities in experimental vitamin A deficiency.. Arch Ophthalmol.

[33] Mutch JR, Richards MB (1939). Keratoconus experimentally produced in the rat by vitamin A deficiency.. Br J Ophthalmol.

[34] West-Mays JA, Cook JR, Sadow PM, Mullady DK, Bargagna-Mohan P, Strissel KJ, Fini ME (1999). Differential inhibition of collagenase and interleukin-1alpha gene expression in cultured corneal fibroblasts by TGF-beta, dexamethasone, and retinoic acid.. Invest Ophthalmol Vis Sci.

[35] Tanzer ML (1973). Cross-linking of Collagen.. Science.

